# Supplementary material for: Enhanced chiroptic properties of nanocomposites of achiral plasmonic nanoparticles decorated with chiral dye-loaded micelles
Source: Nat Commun. 2023 Jan 5;14:81. doi: 10.1038/s41467-022-35699-z (PMC9816153; doi:10.1038/s41467-022-35699-z)
Supplement: Supplementary file 1 — Supplementary Information [file 41467_2022_35699_MOESM1_ESM.pdf]

Supplementary information

Enhanced chiroptic properties of nanocomposites of achiral plasmonic nanoparticles decorated with chiral dye-loaded micelles

Tonghan Zhao[1,4], Dejing Meng[2,4], Zhijian Hu[2], Wenjing Sun[1], Yinglu Ji[2], Jianlei Han[1], Xue Jin[1], Xiaochun Wu[2,3], & Pengfei Duan[1,3]

[1]CAS Key Laboratory of Nanosystem and Hierarchical Fabrication, National Center for Nanoscience and Technology (NCNST), No.11, ZhongGuanCun BeiYiTiao, Beijing 100190, P.R. China.

[2]CAS Key Laboratory of Standardization and Measurement for Nanotechnology, National Center for Nanoscience and Technology (NCNST), No.11, ZhongGuanCun BeiYiTiao, Beijing 100190, P.R. China.

[3]University of Chinese Academy of Sciences, Beijing 100049, P. R. China.

[4]These authors contributed equally: Tonghan Zhao, Dejing Meng.

Correspondence and requests for materials should be addressed to W.X. (email: wuxc@nanoctr.cn) or to P.D. (duanpf@nanoctr.cn)

Contents

1. Figure legends 3
Supplementary Figure 1 3
Supplementary Figure 2 3
Supplementary Figure 3 4
Supplementary Figure 4 5
Supplementary Figure 5 6
Supplementary Figure 6 6
Supplementary Figure 7 7
Supplementary Figure 8 8
Supplementary Figure 9 9
Supplementary Figure 10 9
Supplementary Figure 11 10
Supplementary Figure 12 10
Supplementary Figure 13 11
Supplementary Figure 14 11
Supplementary Figure 15 12
Supplementary Figure 16 12
Supplementary Figure 17 13
2. Table legend 13
Supplementary Table 1 13

1. Figure legends

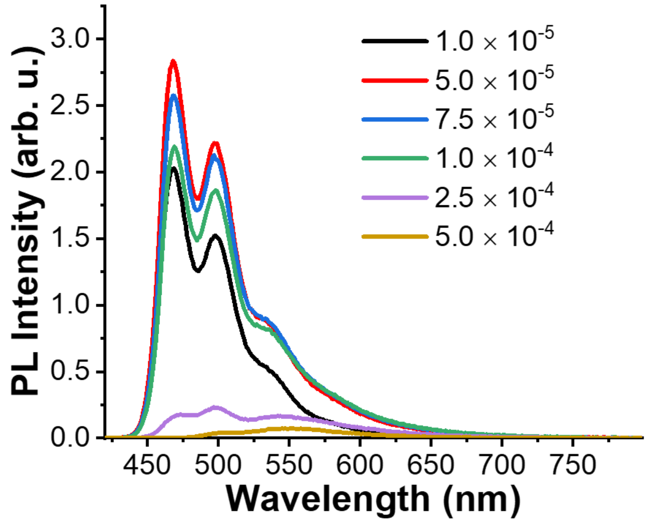

Supplementary Figure 1. Photoluminescence spectra of *R*-1M with different concentrations of *R*-1 in water. [CTAB] = 10^{-2} mol L^{-1}, λ_{ex} = 400 nm. Source data are provided as a Source Data file.

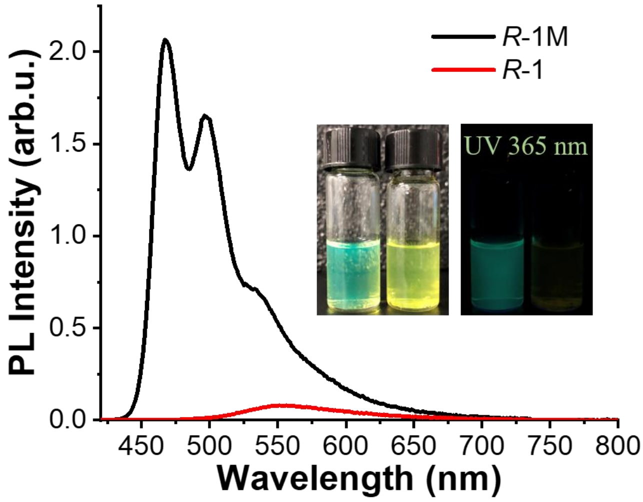

Supplementary Figure 2. Photoluminescence spectra of *R*-1 and *R*-1M in aqueous solution. Inserts were the photographs of these solution under day light (left) and under UV light (right). [*R*-1] = 5×10^{-5} mol L^{-1}, [CTAB] = 10^{-2} mol L^{-1}, λ_{ex} = 400 nm. Source data are provided as a Source Data file.

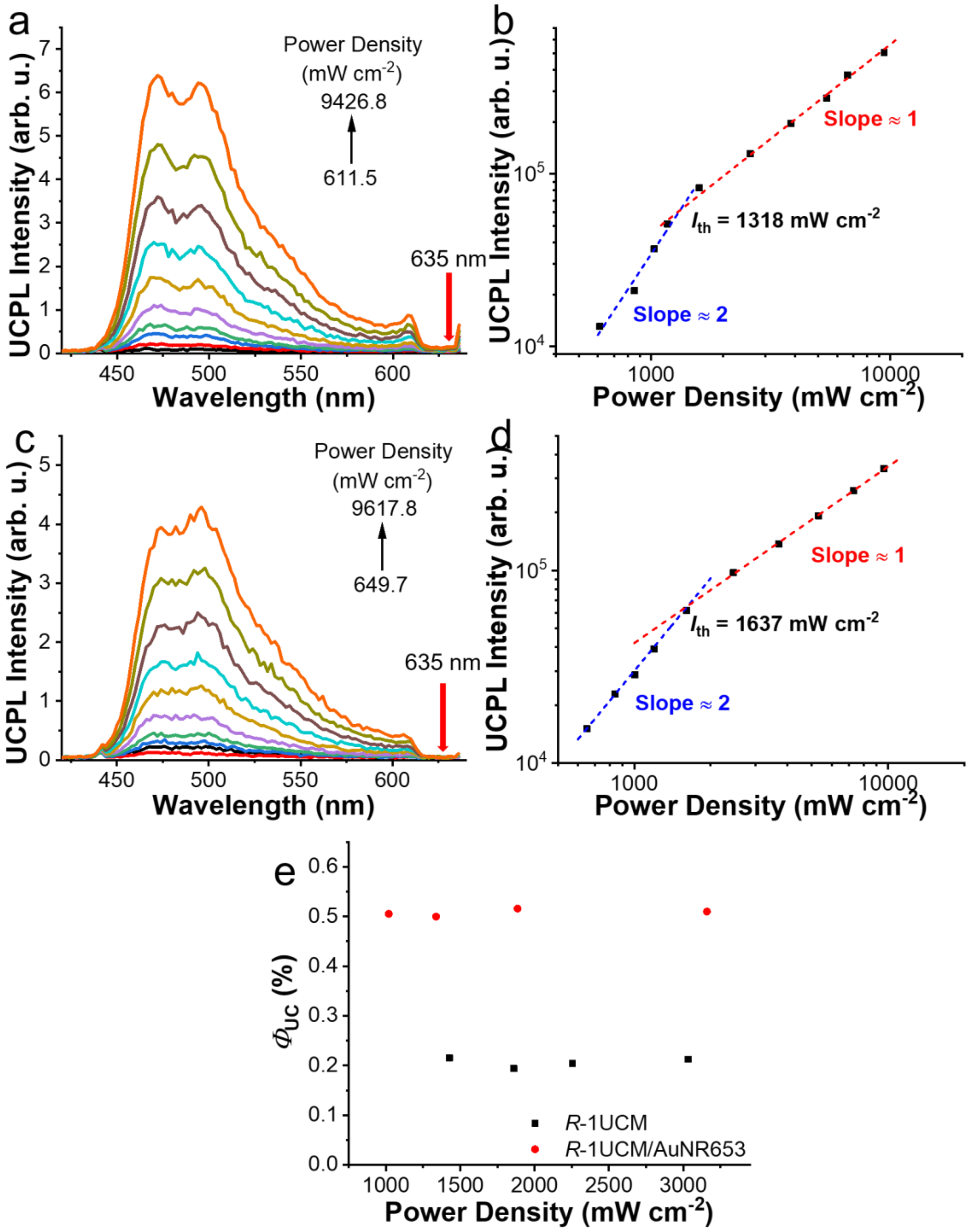

Supplementary Figure 3. Upconverted photoluminescence spectra of (a) *R*-1UCM/AuNR653 composites and (c) *R*-1UCM in deaerated water solution with different excitation power density of 635 nm laser. A 635 nm short pass filter was set between the sample and detector to remove the scattered incident light. Double logarithmic plots of the upconverted photoluminescence intensity of (b) *R*-1UCM/AuNR653 composites and (d) *R*-1UCM as a function of excitation power density. (e) UC quantum yield Φ_{UC} of *R*-1UCM/AuNR653 and *R*-1UCM in aqueous solution as a function of excitation intensity of the 635 nm laser. [*R*-1] = 5 × 10^{-5} mol

L^{-1}, [PdTPBP] = 10^{-5} mol L^{-1}, [CTAB] = 10^{-2} mol L^{-1}, molar ration AuNR653/*R*-1 = $3 \times 10^{-6}/1$. Source data are provided as a Source Data file.

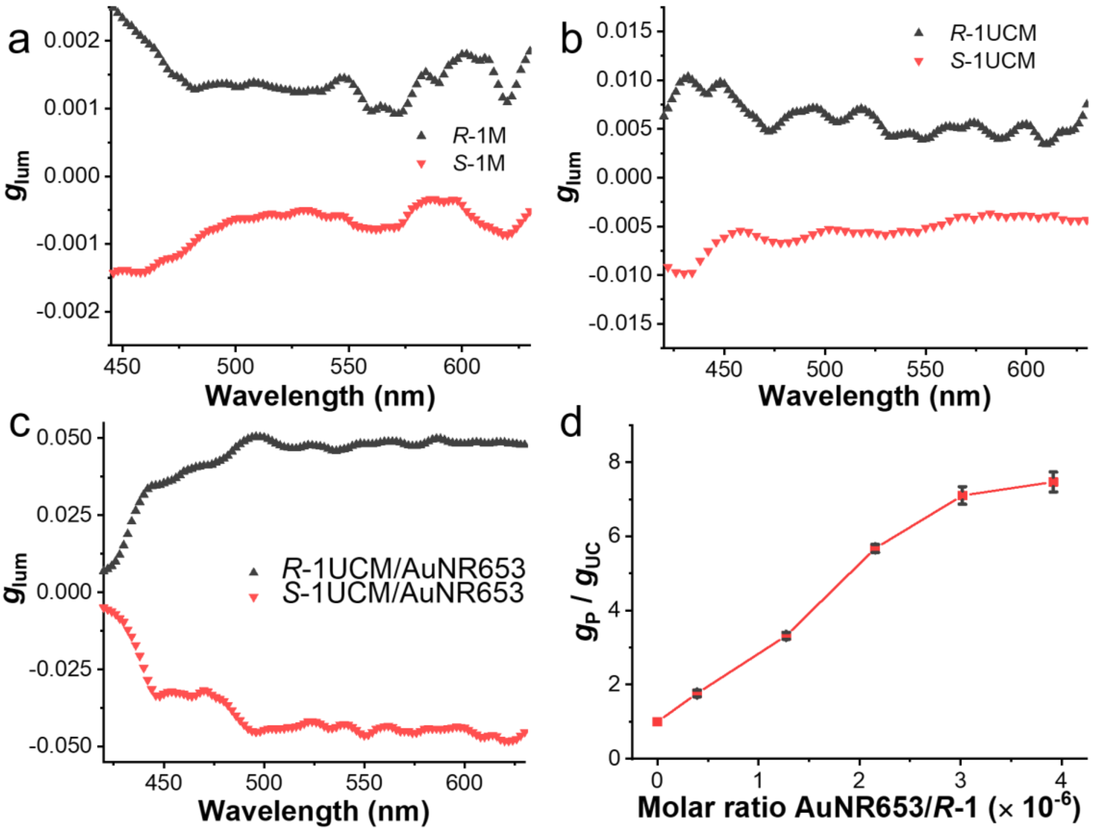

Supplementary Figure 4. Dissymmetry factor g_{lum} values versus wavelength of (a) *R*/*S*-1M, (b) *R*/*S*-1UCM, and (c) *R*/*S*-1UCM/AuNR653. (d) Dissymmetry factor g_{lum} values ratio between *R*-1UCM (g_{uc}) and *R*-1UCM/AuNR653 (g_{p}). [*R*-1] = 5×10^{-5} mol L^{-1}, [PdTPBP] = 10^{-5} mol L^{-1}, [CTAB] = 10^{-2} mol L^{-1}, molar ration AuNR653/*R*-1 = $3.9 \times 10^{-6}/1$. All error bars show mean ± standard deviation. $n = 3$ independent experiments. Source data are provided as a Source Data file.

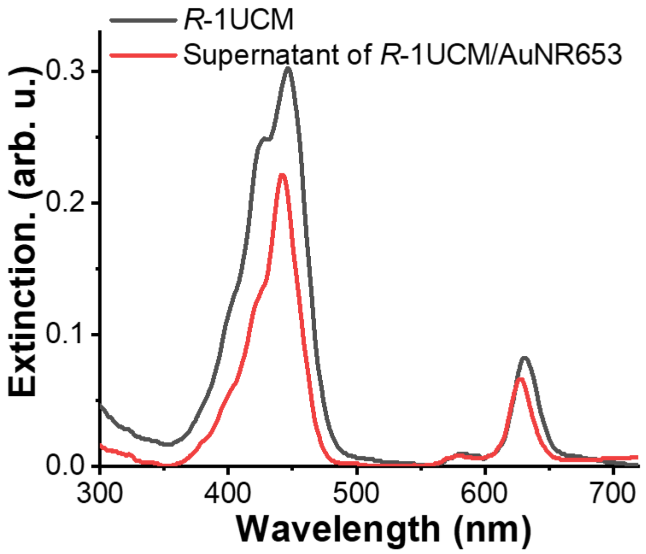

Supplementary Figure 5. Extinction spectra of *R*-1UCM and supernatant of *R*-1UCM/AuNR653 after centrifugation. [*R*-1] = 5 × 10^{-5} mol L^{-1}, [PdTPBP] = 10^{-5} mol L^{-1}, [CTAB] = 10^{-2} mol L^{-1}, molar ration AuNR653/*R*-1 = 3.0 × 10^{-6}/1. Source data are provided as a Source Data file.

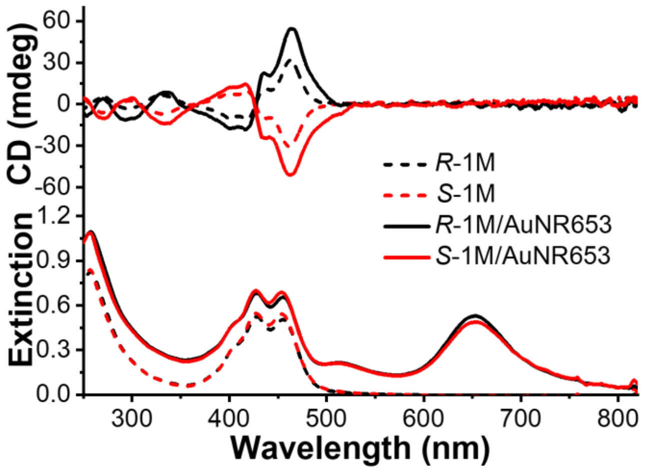

Supplementary Figure 6. CD spectra of *R*/*S*-1MP and *R*/*S*-1MP/AuNR653 composites. [*R*-1] = [*S*-1] = 5 × 10^{-5} mol L^{-1}, [CTAB] = 10^{-2} mol L^{-1}, molar ration AuNR653/*R*-1 = 3 × 10^{-6}/1. Source data are provided as a Source Data file.

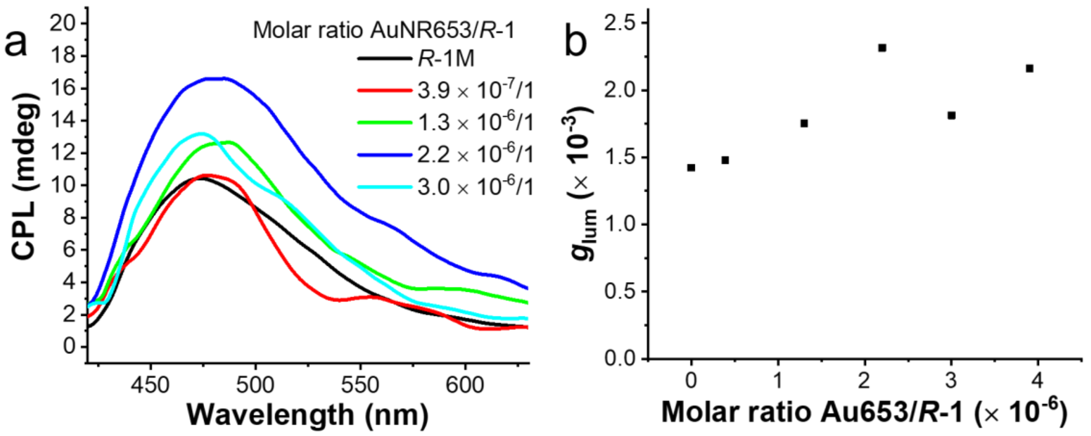

Supplementary Figure 7. (a) CPL spectra and (b) g_{lum} values of *R*-1M and *R*-1M/AuNR653 composites in water. [*R*-1] = 5 × 10^{-5} mol L^{-1}, [CTAB] = 10^{-2} mol L^{-1}, λ_{ex} = 400 nm. Source data are provided as a Source Data file.

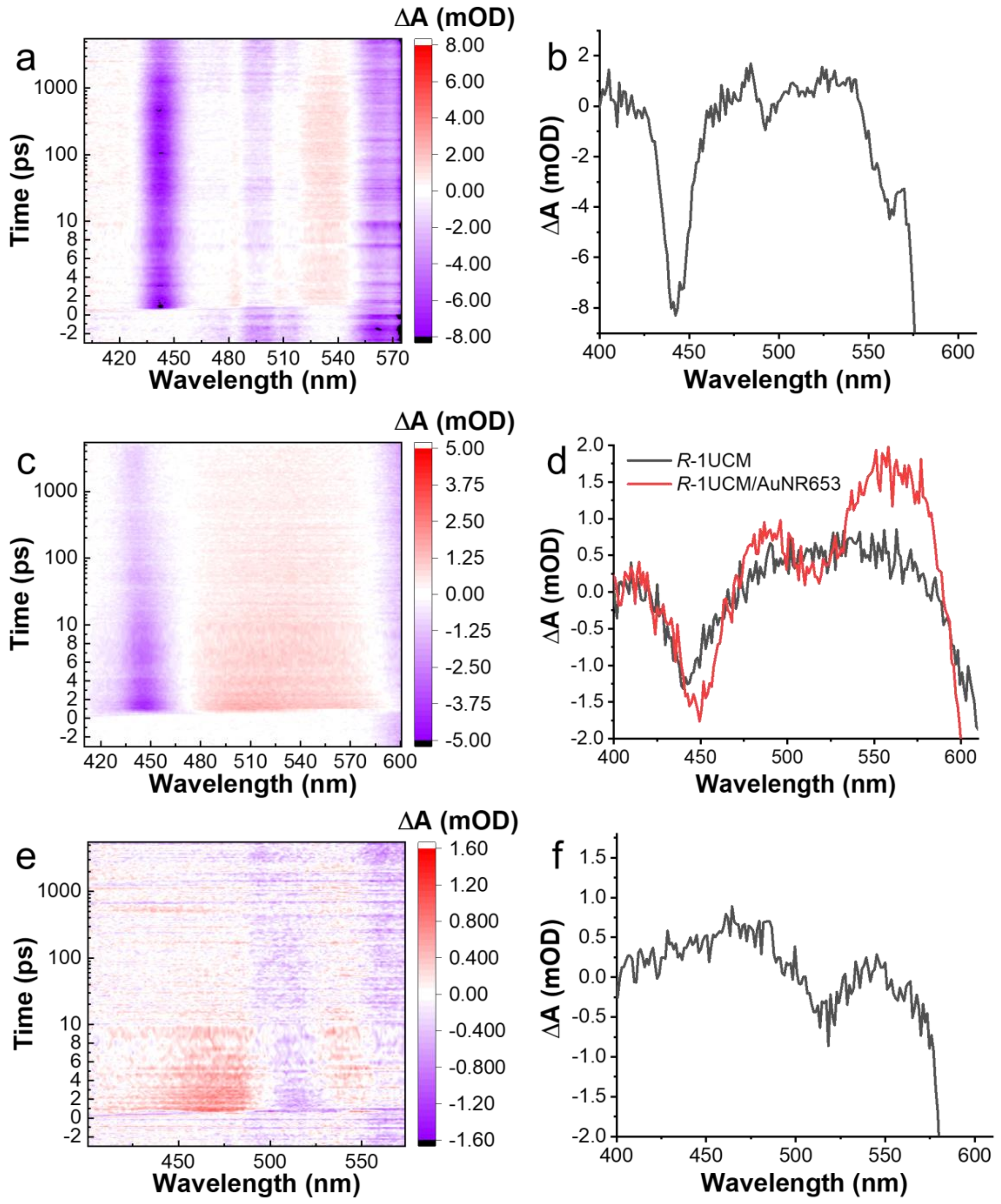

Supplementary Figure 8. Two-dimensional transient absorption spectra of (a) PdOEP micellar aggregation, (c) *R*-1UCM, and (e) AuNR653 in deoxygenated water. Transient absorption spectra of (b) PdOEP micellar aggregation, (d) *R*-1UCM and *R*-1UCM/AuNR653, and (f) AuNR653 in deoxygenated water after excitation at 635 nm, delay time: 1 ps. For *R*-1UCM and *R*-1UCM/AuNR653, delay time is 100 ps. Source data are provided as a Source Data file.

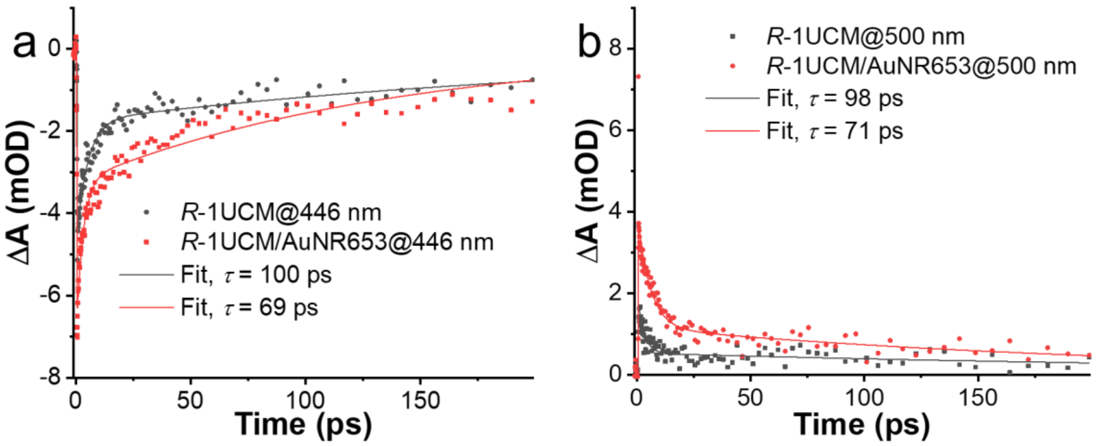

Supplementary Figure 9. Transient kinetics decay curves of *R*-1UCM and *R*-1UCM/AuNR653 monitored at (a) 446 nm and (b) 500 nm, respectively. Source data are provided as a Source Data file.

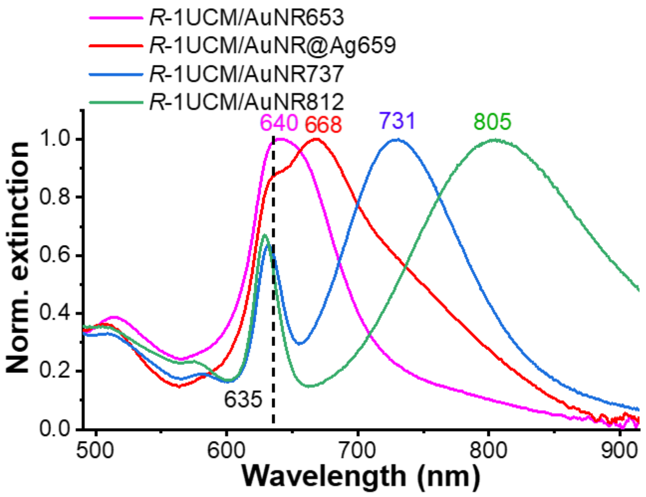

Supplementary Figure 10. Extinction spectra of *R*-1UCM mixed with different nanorods. Source data are provided as a Source Data file.

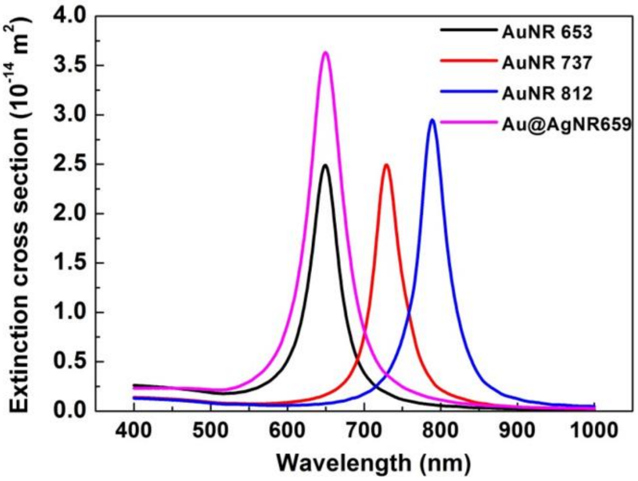

Supplementary Figure 11. Calculated SPR extinction spectra of single AuNRs with different sizes and single AuNR@Ag659. Source data are provided as a Source Data file.

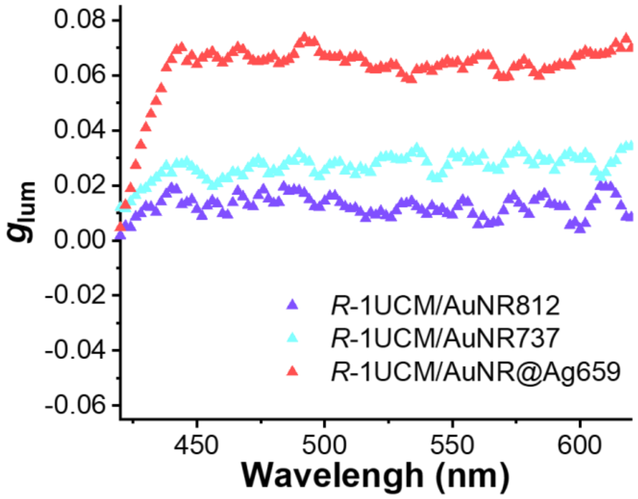

Supplementary Figure 12. Dissymmetry factor g_{lum} versus wavelength of *R*-1UCM coupled with various nanorods. Source data are provided as a Source Data file.

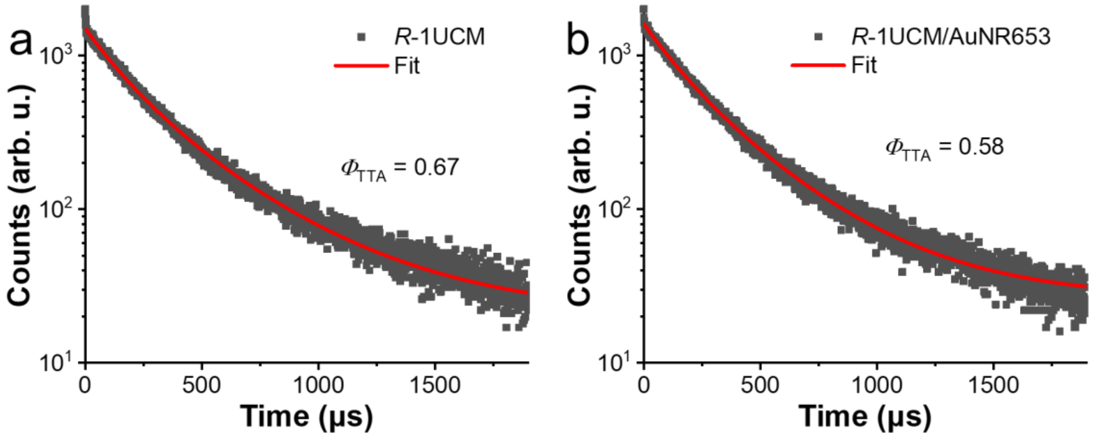

Supplementary Figure 13. The fit of Φ_{TTA} with Equation 2 (red lines) accorded to UC-PL decay of (a) *R*-1UCM and (b) *R*-1UCM/AuNR653 at 476 nm. [*R*-1] = 5 × 10^{-5} mol L^{-1}, [PdTPBP] = 10^{-5} mol L^{-1}, [CTAB] = 10^{-2} mol L^{-1}, molar ratio AuNR653/*R*-1 = 3 × 10^{-6}/1. Excitation source: 635 nm laser with power of 675 mW cm^{-2}. Source data are provided as a Source Data file.

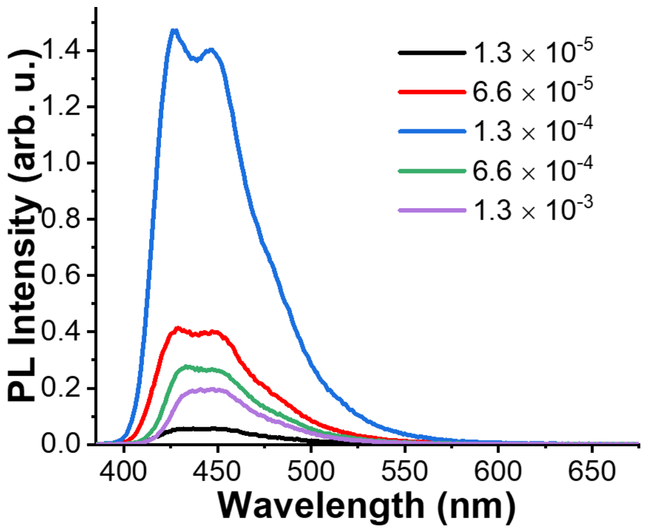

Supplementary Figure 14. Photoluminescence spectra of *R*-2M with different concentrations of *R*-2 in water. [CTAB] = 10^{-2} mol L^{-1}, λ_{ex} = 360 nm. Source data are provided as a Source Data file.

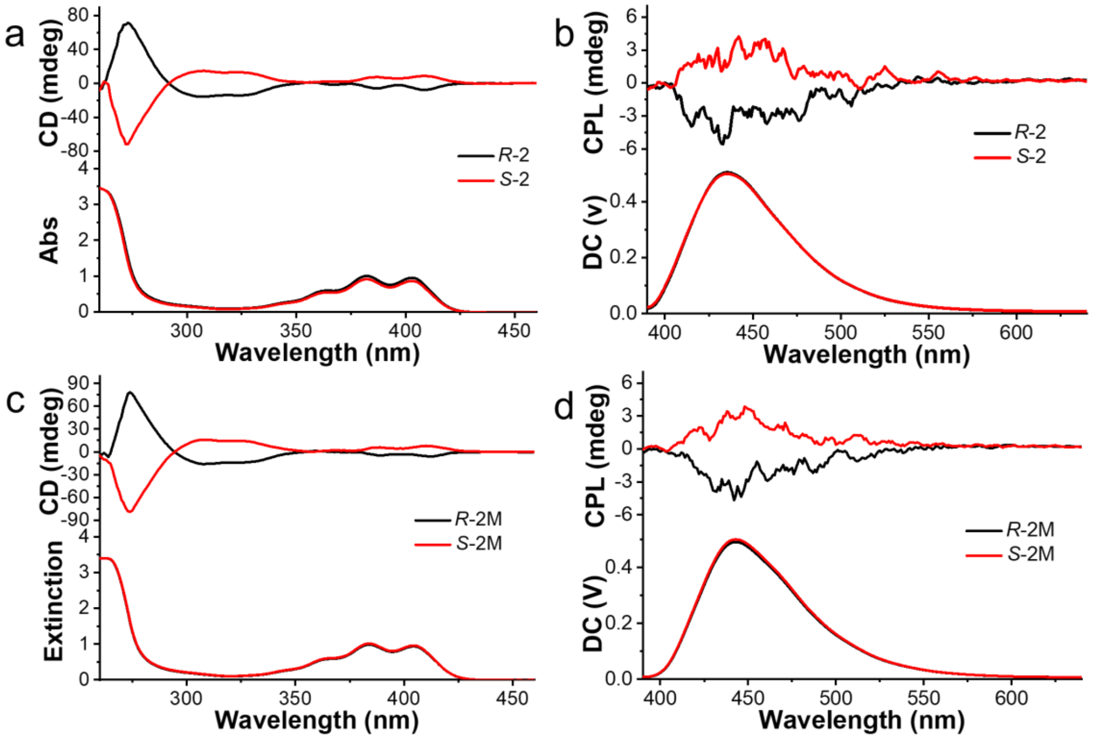

Supplementary Figure 15. (a and c) CD spectra of a) *R*/*S*-2 in THF and c) *R*/*S*-2M in water. (b and d) CPL spectra of b) *R*/*S*-2 in THF and d) *R*/*S*-2M in water. [*R*-2] = [*S*-2] = 1.3 × 10^{-4} mol L^{-1}, [CTAB] = 10^{-2} mol L^{-1}, λ_{ex} = 360 nm. Source data are provided as a Source Data file.

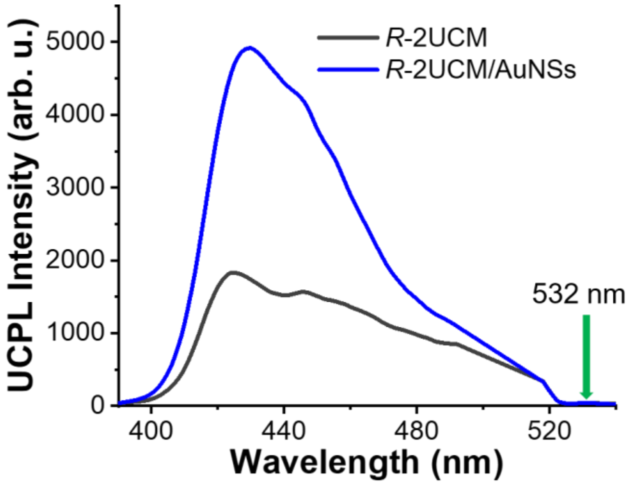

Supplementary Figure 16. Upconverted photoluminescence spectra of *R*-2UCM and *R*-2UCM/AuNSs composites in deaerated water solution under excitation of 532 nm laser with power density of 1650 mW cm^{-2}. A 532 nm short pass filter was set between the sample and detector to remove the scattered incident light. [*R*-2] = 1.3 ×

10^{-4} mol L^{-1}, [PtOEP] = 10^{-5} mol L^{-1}, [CTAB] = 10^{-2} mol L^{-1}, molar ratio AuNSs/*R*-2 = 3×10^{-6}/1. Source data are provided as a Source Data file.

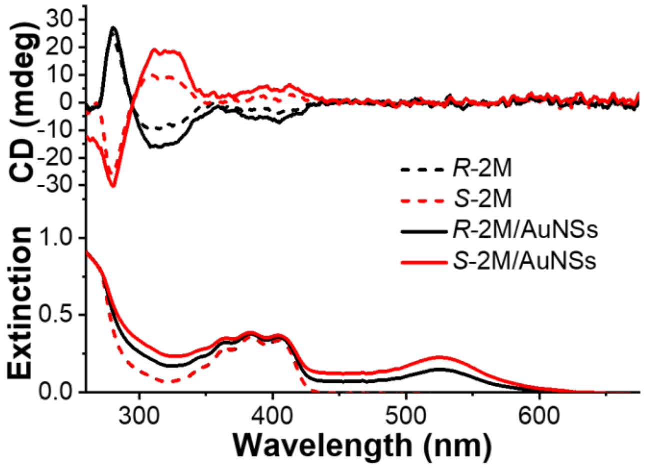

Supplementary Figure 17. CD spectra of *R*/*S*-2M and *R*/*S*-2/AuNSs composites. [*R*-2] = [*S*-2] = 1.3×10^{-5} mol L^{-1}, [CTAB] = 10^{-2} mol L^{-1}, molar ratio AuNSs/*R*-2 = 3×10^{-6}/1. Source data are provided as a Source Data file.

2. Table legend

Supplementary Table 1. Quantum yields of *R*-1 in THF and *R*-1M in water. λ_{ex} = 400 nm.

	R-1	*R*-1M
Φ (%)	35	18
